# Surgical Treatment for a Super-Giant Right Coronary Artery Aneurysm Induced by Kawasaki Disease

**DOI:** 10.21470/1678-9741-2019-0216

**Published:** 2021

**Authors:** Yoshinori Kuroda, Tetsuro Uchida, Azumi Hamasaki, Atsushi Yamashita, Masahiro Mizumoto, Kentaro Akabane, Ai Ishizawa, Mitsuaki Sadahiro

**Affiliations:** 1 Division of Cardiovascular Surgery, Department of Surgery II, Yamagata University Faculty of Medicine, Yamagata, Japan.

**Keywords:** Mucocutaneous Lymph Node Syndrome, Coronary Vessels, Coronary Aneurysm, Tomography, X-Ray Computed

## Abstract

A 32-year-old man diagnosed with Kawasaki disease at the age of three years presented with coronary artery aneurysm (CAA). The aneurysm increased in size, and the patient was referred to our hospital for surgery. Preoperative computed tomography scan showed a super-giant right CAA and giant left CAAs; surgery was performed. The super-giant right CAA was resected, and the ostium of the right coronary artery was closed; then, coronary artery bypass grafting was performed. The left CAAs were not treated surgically because the risk of rupture was low. Here, we describe the successful surgical treatment of a right super-giant CAA.

**Table:** 

Abbreviations, acronyms & symbols	
	**CAA = Coronary artery aneurysm**
	**CABG = Coronary artery bypass grafting**
	**CPB = Cardiopulmonary bypass**
	**CT = Computed tomography**
	**KD = Kawasaki disease**
	**LAD = Left anterior descending artery**
	**LCA = Left circumflex artery**
	**LMT = Left main trunk**
	**RCA = Right coronary artery**
	**SVG = Saphenous vein graft**

## INTRODUCTION

Kawasaki disease (KD) is an acute, self-limiting vasculitis of the whole body in infants and young children^[1]^. Although coronary artery aneurysms (CAAs) are rare, KD may cause them^[1]^. Keyser et al.^[2]^ reviewed data on super-giant CAAs and found that few studies had reported the occurrence of super-giant CAAs measuring > 50 mm due to KD. Furthermore, the best treatment strategy for a giant CAA remains controversial. In this article, we report the successful surgical treatment of a right super-giant CAA, measuring 88 mm, caused by KD.

## CASE REPORT

The patient was a 32-year-old man diagnosed with KD at the age of three years. At the time of diagnosis, a CAA was detected; hence, he underwent regular medical follow-up. Cardiac computed tomography (CT) performed when he was 30 years old revealed a super-giant right CAA measuring 50 mm, and myocardial scintigram revealed an ischemic response in the inferior wall region. However, the giant right CAA was managed conservatively because the necessary surgical procedure could not be performed at the previous hospital. With the continued increase in the size of the aneurysm, he was referred to our hospital for surgery.

**Fig. 1:**
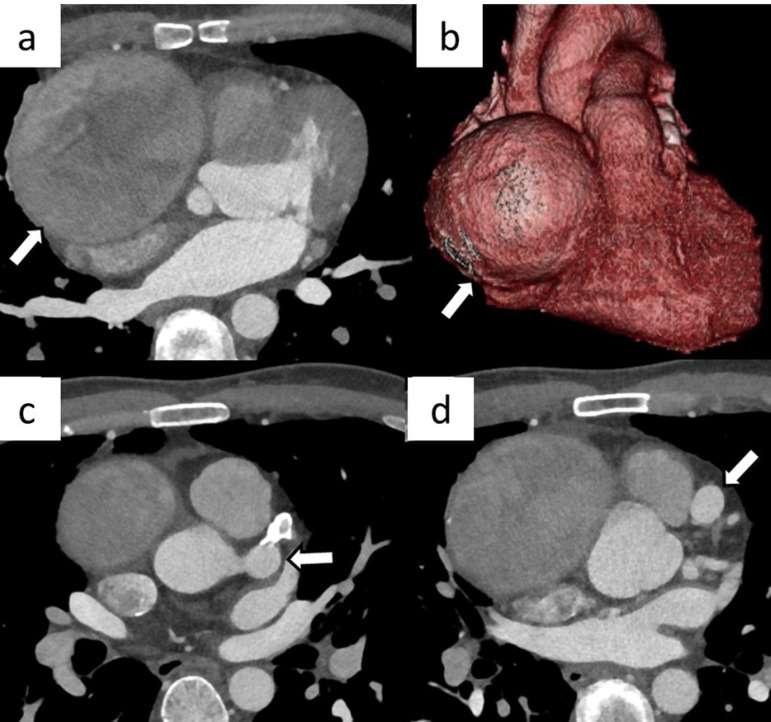
Preoperative cardiac computed tomography scan findings. a, b) Super-giant right coronary artery aneurysm (CAA) (88 mm) arising from the aortic root and displacing the right atrium (arrow). c) Giant CAA (14 mm) in the left main trunk (arrow). d) Giant CAA (12 mm) in the left anterior descending artery (arrow).

A repeat cardiac CT scan showed a super-giant right CAA (88 mm) arising from the aortic root and displacing the right atrium, as well as giant left CAAs in the left main trunk (LMT) (14 mm) and the left anterior descending artery (LAD) (12 mm) (Figure 1). The super-giant right CAA was faintly visualized and no connection to a distal artery was observed on right coronary angiography. On left coronary angiography, the distal portion of the right coronary artery (RCA) was visualized via the collateral circulation from the LAD.

**Fig. 2:**
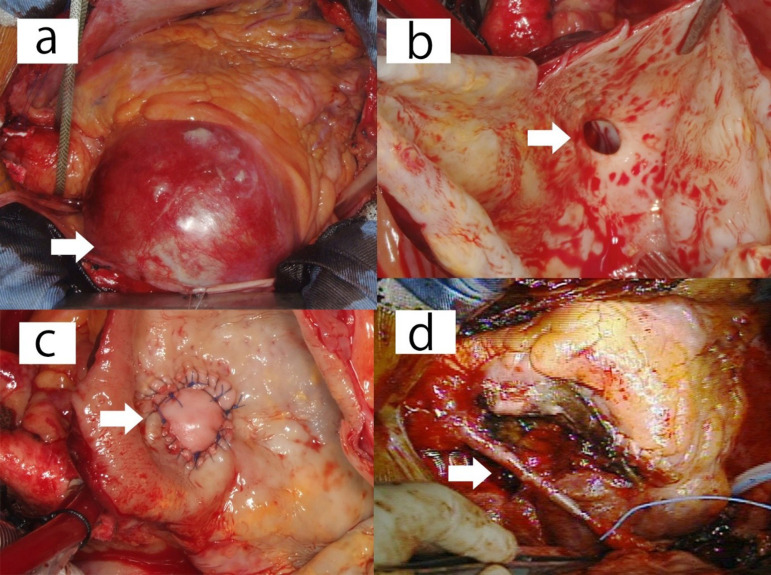
Operative findings. a) Right super-giant coronary artery aneurysm (CAA) at the aortic root, displacing the right atrium (arrow). b) The right super-giant CAA is cut open. An enlarged right coronary ostium (8 mm) is observed at the bottom of the aneurysm (arrow). Communication to the distal coronary artery is not observed. c) The right coronary ostium is closed by interleaving the aortic wall around the ostium with two bovine pericardial patches (arrow). d) Coronary artery bypass grafting to the distal right coronary artery using a saphenous vein graft is performed (arrow).

Median sternotomy surgery was performed. A super-giant right CAA was found at the aortic root, displacing the right atrium (Figure 2A). Cardiopulmonary bypass (CPB) was initiated via the ascending aorta and the superior and inferior vena cava; the patient’s body temperature was maintained at approximately 34ºC. A vent tube was inserted into the left ventricle via the right upper pulmonary vein, and the aorta was clamped. Initially, retrograde cardioplegia was administered; however, ventricular fibrillation occurred, and cardiac arrest was not induced completely. Antegrade cardioplegia was subsequently injected after the first retrograde cardioplegia, which resulted in cardiac arrest. The super-giant right CAA was cut open, and the right coronary ostium at the bottom of the aneurysm was enlarged (8 mm). Communication to the distal coronary artery was not observed (Figure 2B). We closed the right coronary ostium by interleaving with two bovine pericardial patches (Figure 2C). Subsequently, coronary artery bypass grafting (CABG) to the distal RCA was performed using a saphenous vein graft (SVG) (Figure 2D). The left CAAs were not resected because of their small sizes and low risk of rupture. The patient was weaned off CPB easily using 3 µg/kg/min dopamine.

**Fig. 3:**
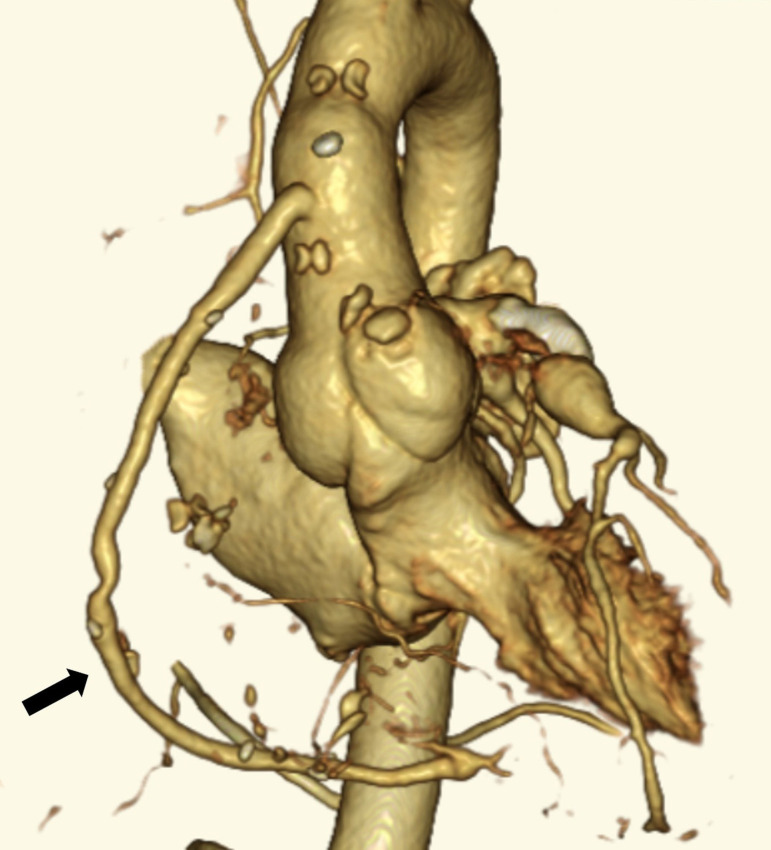
Postoperative cardiac computed tomography scan showing that the saphenous vein graft is patent (arrow) and the right super-giant coronary artery aneurysm has disappeared.

The patient had a good postoperative course. Postoperative cardiac CT revealed that the SVG was patent, and the super-giant right CAA had disappeared (Figure 3). After surgery, warfarin and aspirin were administered to maintain the patency of the SVG and to prevent thrombus formation in persistent CAAs of the left circumflex artery (LCA). Although the left CAAs were still present, the patient remained asymptomatic one year after surgery.

## COMMENTS

CCA is a rare condition defined as coronary artery enlargement wherein the artery diameter is 1.5 times larger than the normal artery diameter. Approximately 0.3-5% of patients who undergo coronary angiography present with CAAs^[4,5]^. A giant CAA is an aneurysm that is four times larger than the normal size or > 8 mm in diameter. Larger CAAs have been reported as super-giant CAAs in a few studies; however, their definition remains unclear^[4-6]^.

Atherosclerosis is the most common cause of CAA. In cases where the atherosclerotic arterial wall is thick and the risk of rupture is low, inflammatory stenosis occurs near the aneurysm. Migration of the thrombus formed in the aneurysm results in the occlusion of the coronary artery, and myocardial infarction then occurs^[3]^. In super-giant CAAs, symptoms due to compression, such as superior vena cava syndrome and fistula formation in the right ventricle, right atrium, or pulmonary artery, occur, and the risk of rupture increases.

The occurrence of super-giant CAAs > 50 mm has been previously reported^[6]^. Similar to the present case, super-giant CAAs (> 85 mm in diameter) due to KD are rare. The most frequent site of CAA is the RCA (52.2%), followed by the LAD, LMT, and LCA, in order of descending prevalence. However, the occurrence of bilateral CAAs is extremely rare (4.5%)^[5]^.

Regarding the treatment of CAA, studies have shown that some clinicians prefer using medications alone, such as anticoagulants or antiplatelets, to prevent cardiac ischemia. Other studies have reported the use of surgical therapy, with good results. Keyser et al.^[2]^ recommended surgery for super-giant CAAs > 50 mm. In most studies, resection of the aneurysm and CABG are performed simultaneously^[2,5,6]^.

CAA caused by KD may decrease in size; hence, we did not perform surgery on the left CAAs that were considered to have a low risk of rupture. In the current case, we chose an SVG for CABG, and both internal thoracic arteries were preserved in case further operation is required for the left CAAs in the future. The indication for operation of CAAs must be determined based on their size and risk of rupture.

**Table:** 

Authors' roles & responsibilities	
YK	Substantial contributions to the conception or design of the work; or the acquisition, analysis, or interpretation of data for the work; drafting the work or revising it critically for important intellectual content; final approval of the version to be published
TU	Drafting the work or revising it critically for important intellectual content; final approval of the version to be published
AH	Substantial contributions to the interpretation of data for the work; final approval of the version to be published
AY	Substantial contributions to the interpretation of data for the work; final approval of the version to be published
MM	Substantial contributions to the interpretation of data for the work; final approval of the version to be published
KA	Substantial contributions to the interpretation of data for the work; final approval of the version to be published
AI	Substantial contributions to the interpretation of data for the work; final approval of the version to be published
MS	Final approval of the version to be published
